# A Population-Based Approach to Study the Impact of PROP Perception on Food Liking in Populations along the Silk Road

**DOI:** 10.1371/journal.pone.0091716

**Published:** 2014-03-13

**Authors:** Antonietta Robino, Massimo Mezzavilla, Nicola Pirastu, Maddalena Dognini, Beverly J. Tepper, Paolo Gasparini

**Affiliations:** 1 Institute for Maternal and Child Health - IRCCS “Burlo Garofolo”, Trieste, Italy; 2 University of Trieste, Trieste, Italy; 3 Condotta Slow Food, Trieste, Italy; 4 Department of Food Science, School of Environmental and Biological Sciences, Rutgers University, New Brunswick, New Jersey, United States of America; Duke University Medical Center, United States of America

## Abstract

Taste is one of the main factors determining food choices. Differences in PROP bitter taste perception have been implicated in individual differences in food preferences and selection. The present study examined associations between, PROP phenotypes, self-reported food liking and TAS2R38 polymorphisms, the major gene implicated in PROP bitterness, in six different populations of the Caucasus and Central Asia, located along the ancient Silk Road. Differences in the distribution of PROP phenotypes across populations were detected, with a higher frequency of super tasters in Tajikistan (31.3%) and Armenia (39.0%) and a higher frequency of non tasters in Georgia (50.9%). While no relationships were observed between PROP phenotypes and food liking using standard statistical tests, we used an approach based on comparison of distance matrices derived from these data. The first matrix compared the food liking ratings of each population to all others pairwise using the Kruskal-Wallis test (at p<0.00063), and the second one compared the distribution of PROP phenotypes across all populations in a similar manner calculating the chi-square statistic as a distance measure. A strong correlation between the two matrices was found (Mantel test: r = 0.67, p-value = 0.03), suggesting that the pattern of food liking across populations was closely related to the distribution of PROP phenotypes. This same relationship was not observed when TAS2R38 genotypes were substituted for PROP phenotypes in this analysis. Our data suggest that a population-based approach utilizing distance matrices is a useful technique for detecting PROP-related differences in food liking and can be applied to other taste phenotypes.

## Introduction

Bitter taste perception is a variable trait both within and between human populations, and large individual differences in responsiveness to bitterness have been well documented [1]. Bitter perception in humans is mediated by a family of 25 TAS2R taste receptors [2]. Among them, the most studied is the *TAS2R38* gene, associated with the ability to taste PTC (phenylthiocarbamide) and PROP (6-n-propylthiouracil) [3]. Approximately 75% of the world’s population are considered “tasters”, and perceive these substances as moderately to intensely bitter. These compounds are weak or tasteless for the remaining 25% of the population, who are considered “non tasters” [4]–[5]. Bartoshuk et al revealed that taster individuals can be divided into two sub-groups: medium tasters, who perceived moderate intensity from PTC/PROP, and super-tasters, who perceived these compounds as extremely bitter. Thus, the population distribution of non tasters, medium tasters and super tasters is approximately 25%, 50% and 25% respectively [6].

Sequence variations in the *TAS2R38* gene produce three amino acid substitutions: A49P, A262V and V291I that define two common haplotypes, namely PAV and AVI. The AVI haplotype (AVI/AVI homozygous individuals) specifies the non taster phenotype, while it was supposed that the PAV haplotype (PAV/PAV homozygous or PAV/AVI heterozygous individuals) specifies the taster phenotype [7]. Although supertasting is typically associated with heightened responses to the bitterness elicited by PROP, TAS2R38 variations cannot explain “general” supertasting more broadly defined as the ability to perceive oral sensations more strongly without regard to PROP status or TAS2R38 polymorphisms [8].

Rare haplotypes (AAI, AAV, PAI, and PVI) have also been observed at a frequency of 1–5% [9], but are mainly found in African populations [10].

PTC and PROP are synthetic compounds, not found in nature, but they are chemically similar to isothiocyanates commonly found in broccoli, cabbage and other bitter-tasting *Brassica* vegetables [11]. The presence of the thiourea group (N-C = S) within these compounds is responsible for their bitter taste. Although the TAS2R38 receptor is also capable of binding non-thiourea substances (e.g., limonin, ethylpyrazine), compounds with theN-C = S moiety are considered the primary ligands for this receptor [12]. Cell-based studies demonstrate that greater PTC binding efficiency and receptor activation is strongly associated with the PAV (taster) form of this receptor [13].

Although variation in *TAS2R38* accounts for 50%–80% of the phenotype [7]–[14], recent data suggest that other genetic and non-genetic factors may play a role. This evidence includes a recently identified SNP (rs2274333) in the gustin (CA6) gene that has been implicated in taste bud growth and maintenance [15]. The presence of the major allele at this location in gustin is associated with greater taste bud densities, which are commonly observed on the tongue of supertasters [16]. Other evidence suggests that differential release of the salivary protein Ps-1 and II-2 that belong to the family of basic Proline-rich Proteins (bPRPs), may play a permissive role in PROP taste bitterness. Moreover, supplementation of Ps-1 to individuals who lack this protein enhances PROP bitterness intensity [16]–[17].

Greater perception of PROP is generally [[18]–[19]–[20]–[21]–[22]–[23]], but not always [24–25–26] associated with dislike and avoidance of *Brassica* vegetables. There are also numerous reports that supertasters dislike bitter foods that do not contain the thiourea group, as well as other foods that produce strong oral sensations such as sweets, added fats, spicy foods and alcoholic beverages [19]–[20]–[21]–[22]–[27]–[28]–[29]. In light of these observations, PROP-tasting has gained attention as general marker for oral sensations and food preferences [18]. This view remains controversial, however, since some studies report no relationship between PROP tasting and general food preferences [25]–[30] and other markers for oral sensations have emerged [31].

Despite these uncertainties, PROP-tasting has shown relationships with food intake and body weight variation that may have import long-term health implications. Specifically, studies showed that non taster women maintained higher body weights [32]–[33]–[34] and consumed more calories and high-fat foods [35]–[36] than supertaster women when offered a variety of foods in a buffet feeding regimen. Preliminary evidence from a mixed-gender, weight-loss intervention showed that almost half (47%) of the obese participants were non tasters as compared to the prevalence rate of 28–32% in the general population [37]. Finally, differences in the risk of colorectal cancer which is mediated, in part, by diet has been reported across TAS2R38 polymorphic groups [38]–[39].

Most population-based studies relating PROP-tasting to food and/or beverage selection have been conducted in Caucasian subjects residing in North America, Australia and Western Europe [19]–[23]–[28]–[40]–[41]. Notable exceptions are the study by Baranowski et al. [42] who studied White, Hispanic and Black children in the U.S.A. and the study by Mennella et al. [43] on African-American and non-Hispanic children and their mothers. Noteworthy works were also conducted in Asian populations [44]–[45]–[46]. There is a wealth of historic data on the diversity of PROP-tasting in different ethnic groups around the globe which shows that the frequency of non-tasting ranges from 3% to >40% worldwide [4].

However, data are sparse on the distribution of *TAS2R38* polymorphisms in these same populations. Some recent investigations addressing this question include a genetic analysis of India-born Asian Indians living in the U.S.A. [47], and a study of genotype-phenotype relationships in a sample derived from West Central and East Africa [10]. However, neither study investigated food liking.

The present study was designed to address this gap in knowledge. Here, we examined relationships among PROP perception, TAS2R38 polymorphisms and food liking in different rural communities from the Caucasus region (Georgia, Armenia and Azerbaijan), Central Asia (Uzbekistan and Kazakhstan) and Tajikistan. Data were obtained as part of the scientific expedition Marcopolo 2010 (www.marcopolo2010.it), whose main goals were to analyse individual differences in the human senses (e.g. taste, smell, hearing, vision) across the Silk Road, a major pathway for cultural, commercial, and genetic exchange between individuals from China and Mediterranean countries for almost 3,000 years.

## Materials and Methods

### Study population

**Figure 1:**
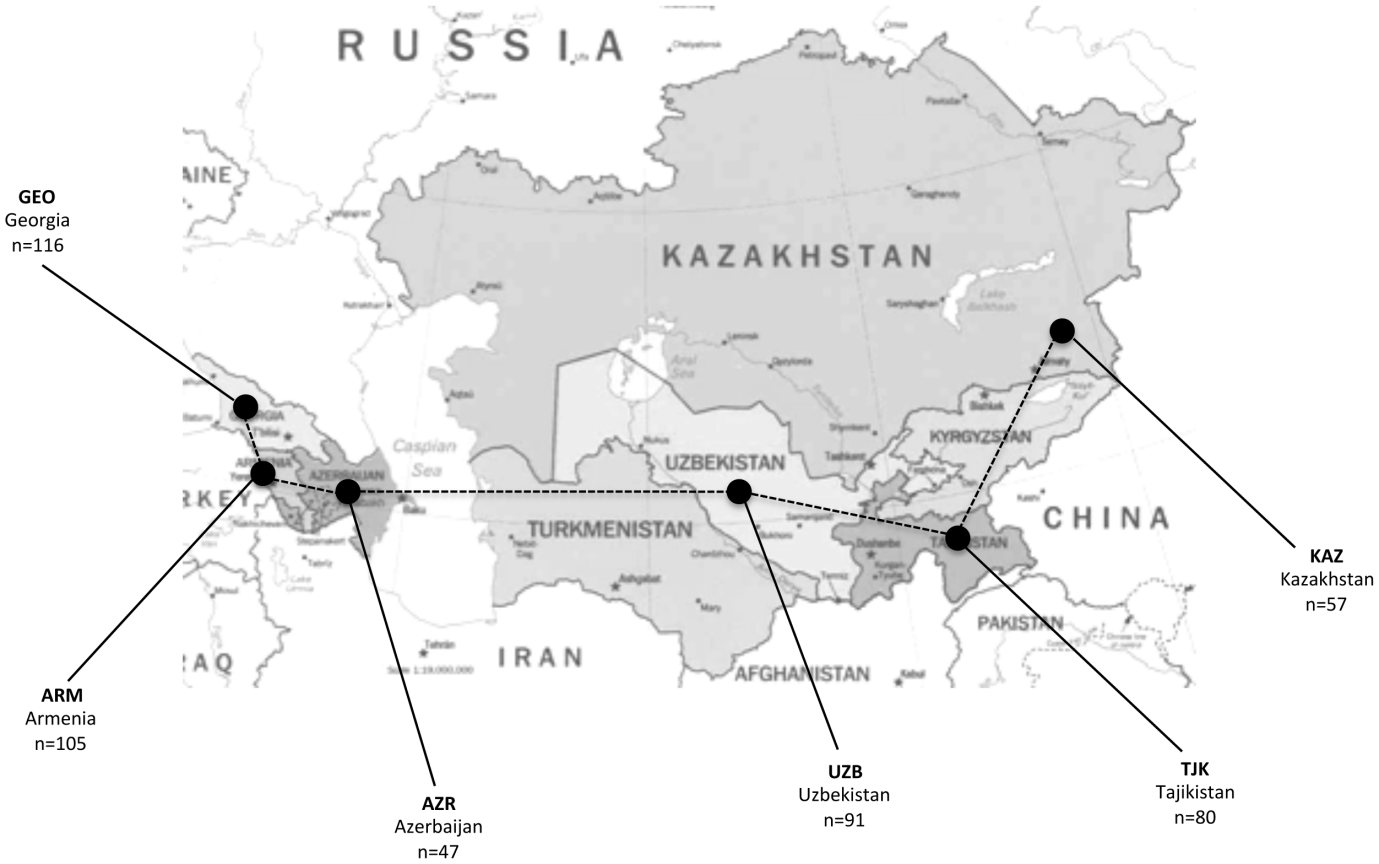
Populations along the Silk Road. Populations analysed (linked by dashed line), their geographical location and sample size.

A total of 496 subjects participated in the study (206 males and 290 females), coming from 20 different communities of six countries in the Caucasus and Central Asia: Georgia, Armenia, Azerbaijan, Uzbekistan, Kazakhstan and Tajikistan (Figure 1). Sample sizes from each of the communities are described in Table S1. All communities belong to the Terra Madre organization (www.terramadre.org). Information, such as age, sex, lifestyle, eating habits, professional activity, smoking and alcohol consumption were collected.

All subjects provided written informed consent before participation. Approval for the research protocol was obtained from the ethical committee of IRCCS-Burlo Garofolo Hospital.

### Genotype analyses

Saliva samples were collected from all participants using the Oragene DNA collection kit and DNA was extracted (DNA Genotek, Ontario, Canada). Three polymorphisms in the TAS2R38 receptor gene (rs1726866, rs10246939 and rs713598) define the genotype. The first two were genotyped with the Omni Express 700k Illumina Chip. The third one was analysed using TaqMan probe-based assays (Applied Biosystems, Foster City, CA, USA).

### PROP tasting

PROP taste intensity was determined in all subjects using a filter paper method previously described [48]. Each subject was given two paper disks, the first one was impregnated with 1.0 mol/l NaCl (VWR Scientific, Bridgeport, NJ), and the second disk was impregnated with 50 mmol/l 6-n-2-propylthiouracil (cat. no. P3755; Sigma-Aldrich, St Louis, MO). The subject was asked to rinse the mouth with bottled water, place the paper disk on the tip of the tongue and rate the intensity of the taste using the labelled magnitude scale (LMS). The subjects were also required to rinse with water between tasting each disk and to wait a minimum 30 s before tasting the PROP disk.

The LMS is a quasi-logarithmic 100-mm scale anchored with the labels ‘barely taste it’, ‘weak’, ‘strong’, ‘very strong’ or ‘strongest imaginable’ oral sensation [49].

For this study the LMS was translated in the local language of each community. In addition, translators verbally defined the label descriptors of the scale to each participant and also instructed him/her to make a mark anywhere on the scale, not only near the descriptors.

Using LMS numerical cut-off scores of <15 and >67, the subjects were classified as super tasters and non tasters, respectively. Medium tasters fell between those two limits (>16 and 67). NaCl ratings were used as a reference standard for classifying subject who gave a borderline rating to PROP. The use of NaCl as a reference standard is based on the observation that super tasters give higher ratings to PROP than NaCl, medium tasters give similar ratings to both, and non tasters give higher ratings to NaCl than to PROP [50]. These procedures were developed and validated in previous studies [48] and have been used in numerous investigations in English-speaking and non-English speaking populations followed in our previous studies [34–51–52].

### Food Liking Questionnaire

Participants completed a 79-item food liking questionnaire that was based on an instrument used in a previous study [51] and supplemented with foods specific to the diets of the communities we studied. The original questionnaire focused on bitter-tasting, strong-flavored and high-fat foods that had been associated with PROP status in our previous studies [21]–[51]. The selection of the supplemental foods was based on a survey conducted by collaborators from the Terra Madre organization who carried out a preliminary survey on the local foods consumed by these populations [53]. The questionnaire assessed general food likes and dislikes (e.g. garlic, milk, banana, orange juice). It was administered in the local language of each community by translators who were familiar with the local culture.

Subjects rated their liking of each item on a 5-point scale ranging from “like extremely” (score 5) to “dislike extremely” (score 1). The option “never tasted” was also included.

### Statistical analysis

The Chi-square test was used to examine the association between *TAS2R38* genotypes and PROP status for the whole cohort. Chi-square tests were also performed to determine whether the relationship between *TAS2R38* genotypes and PROP status differed among the populations tested. Correspondence Analysis [54] was also applied to the two-way contingency table of PROP status and participants’ country of residence to obtain a graphical representation of the relationship between the two variables.

Considering the potential relationship between PROP perception and food liking reported in the existing literature [18–19–20–21–28–29], analysis of covariance (ANCOVA) was performed to determine the influence of PROP taster status and *TAS2R38* genotypes on liking of each food. This analysis was applied to the entire cohort and to each population separately. Sex and age were used as the covariates. Due to the large number of comparisons, statistical significance was set at p<0.00063, following Bonferroni correction (p = 0.05/ 79 foods).

In addition, the foods were grouped using the same general categories as in Ullrich et al. [21] and the ANCOVA calculations described previously were repeated. The food groups included fruits (strawberries, lemons, orange juice), vegetables (artichokes, spinach, turnip, cooked carrots, asparagus, fava beans, cabbage), alcohol (red wine, white wine, vodka, brandy, beer), condiments (olives, sardines, onion, garlic, kilka, adgika, chilli pepper), sweets (ice cream, cake, sweet ricotta, biscuits, biscuits with cream, jam, honey, milk chocolate). The mean number of foods within each food group was calculated for each subject and was used for the analyses.

We also sought to determine if variations in food likes and dislikes across populations were related to the distribution of PROP phenotypes or *TAS2R38* genotypes. To accomplish this task, a series of data matrices were constructed. First, the Kruskal-Wallis test was performed (at p<0.00063) comparing the food liking of each population to all others, pairwise. The number of foods that showed statistically significant differences between population pairs were tallied and entered into a distance matrix. Higher values indicated dissimilar patterns (large distances) in food liking between populations, and lower values indicated similar patterns (small distances) between them. For example, if the pair-wise difference between two populations was high, these two populations had many differences in food liking. On the contrary, if the pair-wise difference was small, the two populations shared similar food liking responses.

In order to describe the phenotypic dissimilarities in bitter perception between populations, we created another distance matrix. Here, we calculated the chi-square statistic (as a distance measure) between phenotypic groups (non taster, medium taster and super taster) for each population, pairwise. Here, higher values represent a large difference (i.e., distance) in PROP bitterness between population pairs, and lower values represent a small difference in bitterness between population pairs. The data inputs and procedures for this analysis are similar to those of multiple correspondence analysis (MCA) [55] where data are categorical rather than continuous.

In order to assess possible bias due to the differences in sample size between populations, we performed a bootstrap analysis [56]. We constructed a series of distance matrices by repeatedly (1000 times) sampling 47 individual (the n of the smallest population) from each population. We compared each distance matrix built after bootstrapping with the original one (built using the full dataset) and found a high correlation between them (r>0.9), showing that differences in sample size did not affect our results.

Then, we calculated the F_ST_ (Fixation Index) [57] to estimate genetic differences between populations for the SNPs which define TAS2R38 haplotypes. We also constructed a matrix of F_ST_ values using the whole genome (∼356,000 SNPs) to obtain a global estimate of genetic diversity in our sample. Pairwise F_ST_ was performed using the R package Adegenet v1.3-4 [58].

Finally, the Mantel test [59] was used to determine the (dis)similarities between distance matrices. The Mantel r statistic is a standardized Pearson correlation coefficient calculated following random rearrangement of the data matrices across multiple permutations. 1000 iterations were used for a critical cut-off value of p<0.05.

## Results and Discussion

### PROP phenotypes and haplotypes

All 496 individuals were tested for PROP taste intensity. The distribution of PROP status in each population was analysed and is shown in Table 1. In the overall sample 37.0% of individuals were non tasters, 40.0% were medium tasters and 23.0% were super tasters. Interestingly, the distribution of phenotypes varied among the populations (X-squared = 42.1077, p-value = 7.1e-06). In particular, the prevalence of non tasters was higher in Georgia (50.9%) as compared to other populations, while the proportion of super tasters was higher in Armenia (39.0%) and Tajikistan (31.3%) relative to the other populations.

**Table 1:** Distribution of PROP phenotype by sex and population.

	PROP phenotype		
	NT	MT	ST
**All** (n = 496)	37.0%	40.0%	23.0%
**Sex**			
*Males* (n = 206)	44.2%	41.7%	14.1%
*Females* (n = 290)	32.1%	39.3%	28.6%
**Population**			
*Georgia* (n = 116)	50.9%	38.8%	10.3%
*Azerbaijan* (n = 47)	38.3%	46.8%	14.9%
*Uzbekistan* (n = 91)	40.7%	40.7%	18.6%
*Kazakhstan*(n = 57)	31.6%	50.9%	17.5%
*Tajikistan* (n = 80)	36.2%	32.5%	31.3%
*Armenia* (n = 105)	22.0%	39.0%	39.0%

NT = non taster, MT = medium taster, ST = super taster.

**Figure 2:**
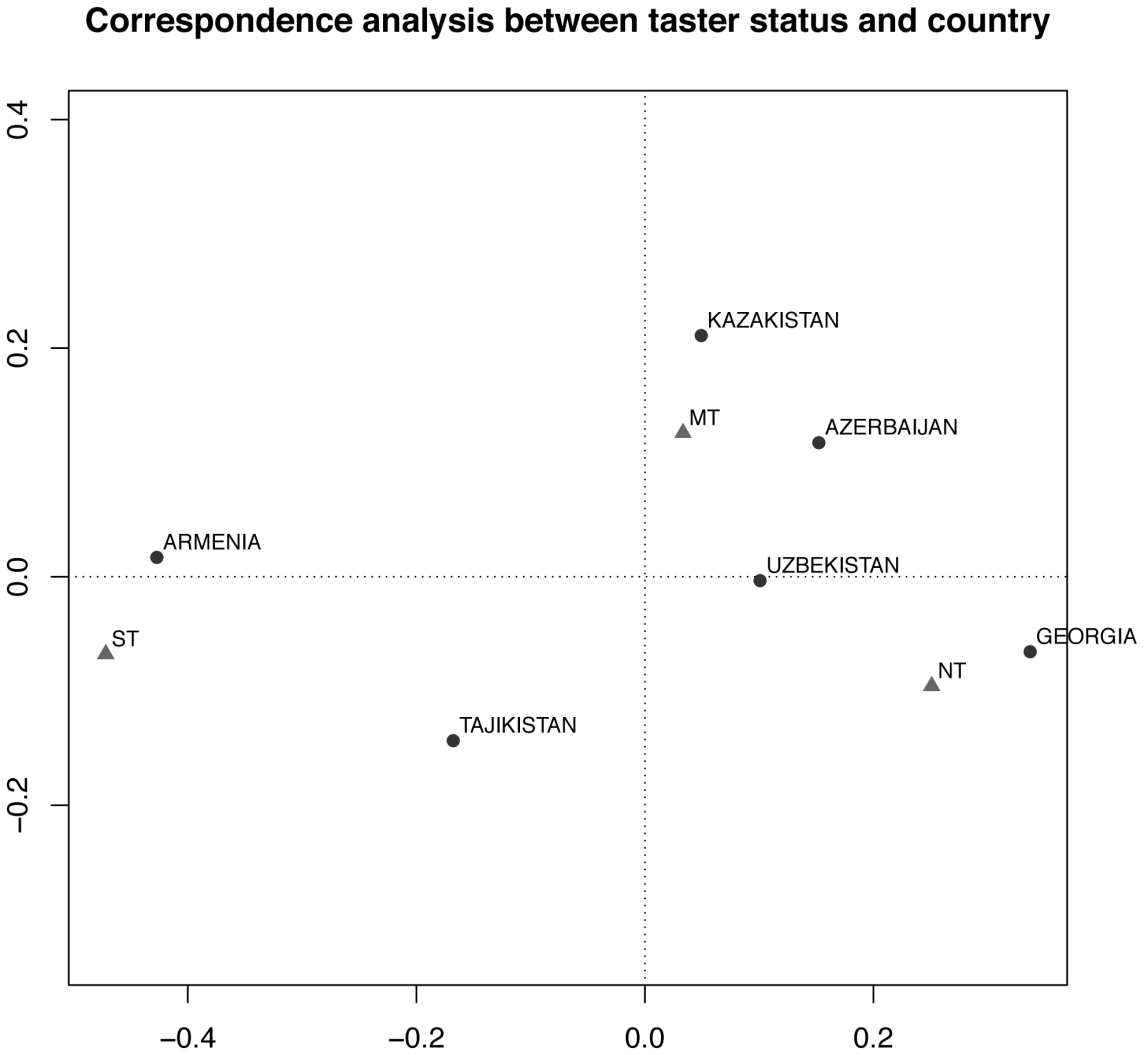
Correspondence analysis between taster status and country. Correspondence Analysis between taster status and country shows the relationship between them. In particular, super taster status corresponds to Armenia and Tajikistan populations, non taster status to Georgia and medium taster status to Azerbaijan, Kazakhstan and Uzbekistan. Circles and triangles represent the country and the PROP status respectively. NT = non taster, MT = medium taster, ST = super taster. Country accounted for the majority (87.3%) of variance and taster status accounted for 12.7% of variance in the model.

Correspondence Analysis revealed the relationships among the populations living in different countries with respect to PROP phenotype. In agreement with the univariate analyses, Georgia was highly associated with the non taster phenotype while Armenia was closely associated with the super taster phenotype. Furthermore, medium tasters were highly represented in the cluster of populations consisting of Azerbaijan, Uzbekistan and Kazakhstan. Tajikistan was distinct from the other groups (having relatively equal frequencies of the three taster phenotypes), although it was more closely associated with the super taster phenotype, in accordance with the high prevalence of super tasters in this population (Figure 2).

These findings do not agree with a simple geographical explanation for the pattern of PROP phenotypes across populations. In particular, the phenotype differences between the populations of Armenia and Georgia were totally unexpected, because these two countries are closely located and have a long standing tradition of cultural and political exchange dating back to the Middle Ages, when the two countries were allied against the Muslim empire [60].

Differences in age, gender and smoking can influence PROP phenotypes [6–34–61–62]. However, these factors did not explain the variability across the populations studied here since our analyses adjusted for these factors. These data support recent findings suggesting that other genetic loci or non-genetic factors contribute to PROP tasting [15]–[17] and efforts to identify and fully characterize these factors should be an on going goal.

In contrast to the phenotypic differences observed among populations, we found no differences in *TAS2R38* haplotypes across populations (X-squared = 8.1822, p-value = 0.611) (Table 2). The AVI/AVI, AVI/PAV and PAV/PAV diplotypes accounted for 24.9%, 48.0% and 27.1%, respectively, of the overall sample, in agreement with the allelic frequencies typically reported in Caucasian populations [7]. As expected, there was a strong association between TAS2R38 diplotypes and PROP phenotypes (X-squared = 151.4019, p-value <2.2e-16). In the entire sample 82.9% of AVI/AVI homozygous individuals were non tasters, compared to 11.4% who were medium tasters and 5.7% who were super tasters. As expected, PAV/PAV homozygous and PAV/AVI heterozygous subjects were mainly medium or super tasters. We observed a similar correspondence between genotypes and phenotypes in each population. These data are reported in Table S2.

**Table 2:** Distribution of TAS2R38 haplotype by sex and population.

	TAS2R38 haplotype		
	AVI/AVI	PAV/AVI	PAV/PAV
**All** (n = 496)	24.9%	48.0%	27.1%
**Sex**			
*Males* (n = 206)	24.8%	48.5%	26.7%
*Females* (n = 290)	25.0%	47.6%	27.4%
**Population**			
*Georgia* (n = 116)	33.7%	42.2%	24.1%
*Azerbaijan* (n = 47)	15.2%	56.5%	28.3%
*Uzbekistan* (n = 91)	22.2%	51.1%	26.7%
*Kazakhstan*(n = 57)	24.6%	49.1%	26.3%
*Tajikistan* (n = 80)	23.8%	47.6%	27.8%
*Armenia* (n = 105)	22.9%	47.6%	29.5%

### PROP phenotype and food liking

The relationship between PROP phenotype and liking for each food on the food liking questionnaire was examined for the entire cohort, and separately for each population, and no associations were found. A list of all foods with mean and standard deviation in the overall sample and in each population is reported in Table S3.

In addition, no relationship was revealed between PROP status and food preference groups. Data are shown in Table S4.

These same analyses were repeated for *TAS2R38* haplotypes, and the outcome was the same; no associations were found.

### Multi-dimensional analyses of food liking

**Figure 3:**
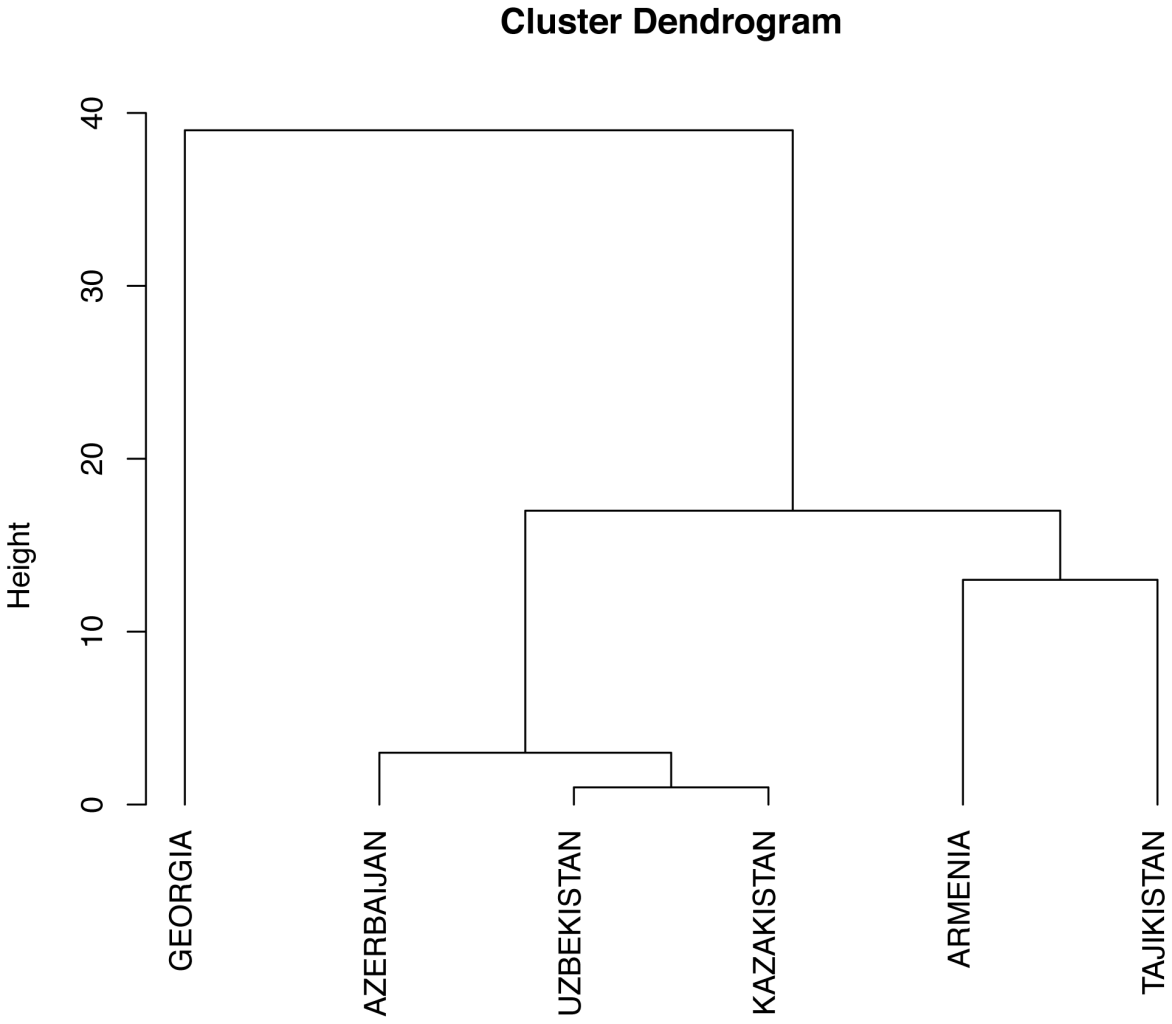
Dendrogram based on differences in food preferences between populations. Three groups are clear from the dendrogram: one composed by Georgia, the second one by Uzbekistan Kazakhstan and Azerbaijan and the third one by Armenia and Tajikistan.

A distance matrix describing the differences in food liking across the populations was constructed, and is graphically presented as a dendrogram in Figure 3. The figure shows three different groups: the first one composed only of Georgia, the second one composed of Uzbekistan, Kazakhstan and Azerbaijan and the third composed of Armenia and Tajikistan. It is clear that countries do not group according to geography, especially in the case of Armenia and Tajikistan.

We then determined if the PROP bitterness phenotypes could explain the observed clustering. Thus, we compared the two distance matrices (the PROP phenotype on one hand and the food liking on the other) and found a strong positive correlation between them (Mantel test: r = 0.67, p-value = 0.003). The results of the Mantel test between each pair of distance matrices are summarized in Table 3.

**Table 3:** Mantel test results between distance matrices analyzed.

	Geography	GenomicFst	TAS2R38Fst	PROP Status
**Genomic Fst**	**0.79**			
**TAS2R38 Fst**	–0.04	–0.04		
**PROP Status**	–0.32	–0.37	–0.18	
**Food liking**	0.20	0.02	–0.30	**0.67**

In bold are reported significant correlations.

In addition, distance matrices and their graphical representation are supplied in supplementary materials (Table S5 and Table S6, Figure S1).

We also tested if the *TAS2R38* gene was associated with these groupings, and found no evidence of correlation (correlation = 0.02, p-value = 0.3) between the distance matrix of food liking and the matrix of genetic distance based on *TAS2R38*. In addition no correlation was found using the distance matrix based on the whole genome using ∼356,000 SNPs.

These results have two important implications. First, they show that differences in food liking among populations strongly correlate with PROP taster status but not with *TAS2R38* genotypes. This finding supports the view that polymorphisms in *TAS2R38* primarily define the ability to taste PROP, but also recognizes that this gene is pleiotropic and influences multiple phenotypic traits such as the perception of non-thiourea, bitter and non-bitter tastes, other oral sensations, food liking, and downstream effects such as dietary behaviour and weight status [15]–[18]–[34]. We acknowledge, however, that PROP status may be one of several markers for chemosensory perceptions [31], and multiple markers may be involved. Moreover, post-receptor differences in peripheral signalling to the central nervous system (CNS) may also play a defining role in chemosensory responses [8]–[63]. A better understanding of all these mechanisms may be required to fully capture the depth and breath of human chemosensory experiences, and their influence on food selection.

Second, we did not observe any direct relationships between geography and the distribution of *TAS2R38* haplotypes or between geography and food liking in the populations we studied. Our findings differ from those of Pemberton et al. [47] who studied *TAS2R38* haplotypes in Asian Indians born in 15 geographic regions across India. They found that haplotype frequencies varied along a latitudinal cline with more tasters in the northern groups and more non tasters in the southern groups. Although Pemberton et al. [47] did not study food liking, it is intriguing that hot spices, like chilli pepper are more frequently consumed in southern India [64] in the same areas where non tasters predominate. Given the critical role of geography and climate in shaping the genetic features of world populations [65], we can only speculate that the geographical and ecological barriers to genetic and cultural exchanges in the groups residing in India along a north-south gradient were more formidable than those operating along the Silk Road which has been an east-west corridor for such exchanges for thousands of years.

However, asymmetrical gene flow and the availability of different crops could also be responsible for variability in genetic features across populations [66]. In addition this study was also limited by the sample size in each population.

These aspects can represent the limitations of the present study, therefore future studies involving a deeper analysis of other genes and environmental variables could further elucidate population differences in taste responses and food liking.

In conclusion, we used a population-based approach in which we exploited taste phenotypic differences among populations to reveal differences in food liking patterns across populations that could not be detected using standard methods. This approach, based on comparisons between distance matrices, can be applied to different population groups around the globe to obtain a comprehensive view of the role of PROP tasting in food preferences as well as to explore the role of novel taste-related traits in food choice.

## Supporting Information

Figure S1 **Multidimensional scaling of PROP status (a) and food preferences (b) matrices.**
(TIF)

Table S1 **Examined populations and their sample sizes.**
(DOC)

Table S2 **The percentage of AVI/AVI, PAV/AVI and PAV/PAV subjects in the groups of NT (non taster), MT (medium taster) and ST (super taster) for each population.**
(DOC)

Table S3 **Mean and standard deviation of each food included in the food liking questionnaire in the overall sample and in each population.**
(DOCX)

Table S4 **Mean and standard deviation of food liking groups in each population.**
(DOCX)

Table S5 **Pair-wise distance matrix based on food liking.**
(DOCX)

Table S6 **Pair-wise distance matrix based on PROP status.**
(DOCX)
