# Supplementary figures and images for: A Population-Based Approach to Study the Impact of PROP Perception on Food Liking in Populations along the Silk Road
Source: PLoS One. 2014 Mar 13;9(3):e91716. doi: 10.1371/journal.pone.0091716 (PMC3953580; doi:10.1371/journal.pone.0091716)

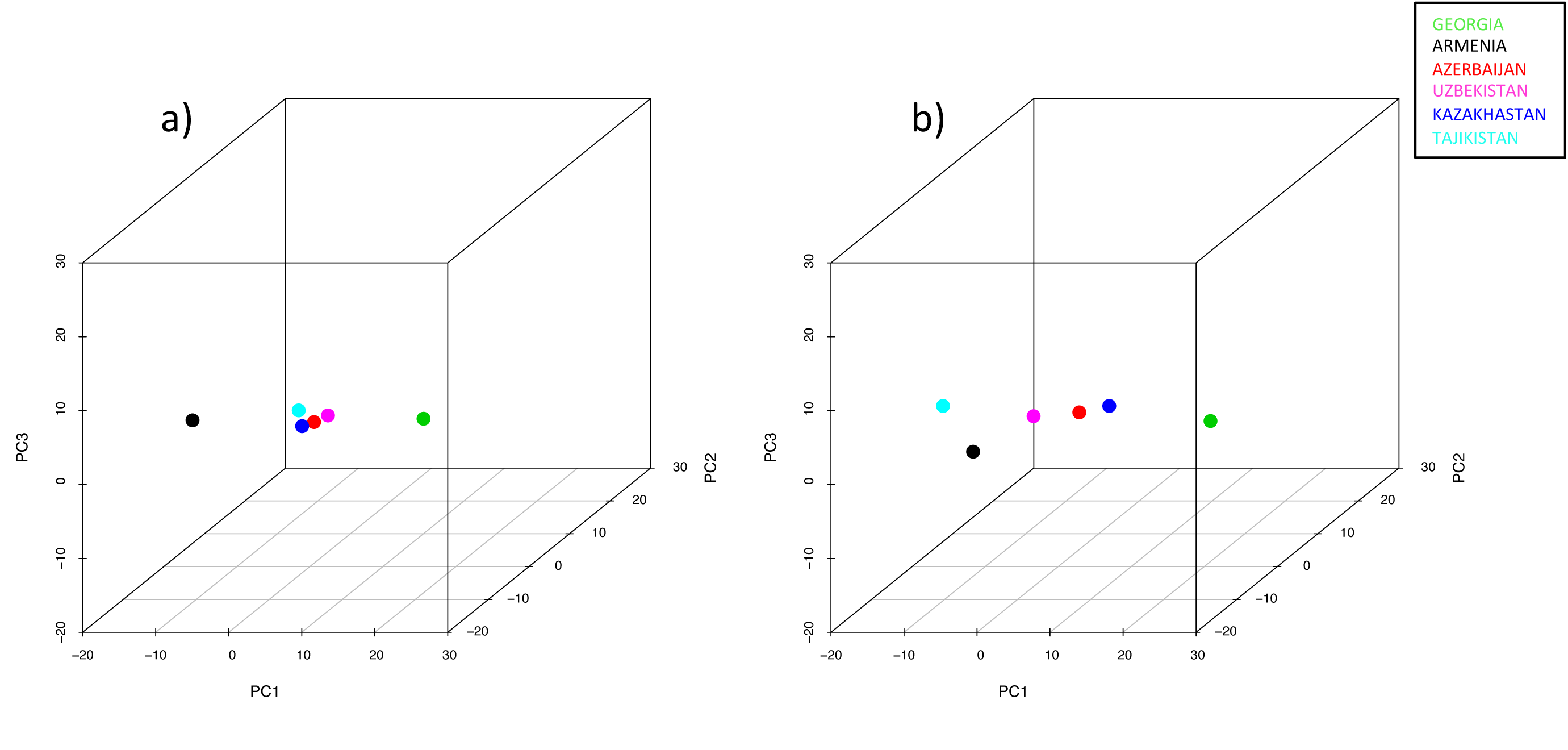

Supplement: Figure S1 — Multidimensional scaling of PROP status (a) and food preferences (b) matrices. (TIF) [file pone.0091716.s001.tif]
