# Supplementary material for: A Population-Based Approach to Study the Impact of PROP Perception on Food Liking in Populations along the Silk Road
Source: PLoS One. 2014 Mar 13;9(3):e91716. doi: 10.1371/journal.pone.0091716 (PMC3953580; doi:10.1371/journal.pone.0091716)
Supplement: Table S1 — Examined populations and their sample sizes. (DOC) [file pone.0091716.s002.doc]

| **Country** | **Community** | **Sample Size**  **(n)** |
| --- | --- | --- |
| *Georgia* |  | 116 |
|  | Imereti | 58 |
|  | Kakheti | 44 |
|  | Mtskheta-Mtianeti | 14 |
| *Azerbaijan* |  | 47 |
|  | Sis | 22 |
|  | Ismailly | 25 |
| *Uzbekistan* |  | 91 |
|  | Karshi | 37 |
|  | Bukhara | 42 |
|  | Tashkent | 12 |
| *Kazakhstan* |  | 57 |
|  | Alga | 29 |
|  | Almaty | 28 |
| *Tajikistan* |  | 80 |
|  | Kalaikhum | 19 |
|  | Khorog | 32 |
|  | Rushan | 13 |
|  | Shing | 16 |
| *Armenia* |  | 105 |
|  | Chambarak | 34 |
|  | Deprabak | 10 |
|  | Gavar | 15 |
|  | Martumi | 16 |
|  | Yeghvard | 20 |
|  | Yerevan | 10 |

**Table S1. Examined populations and their sample sizes**
