# Supplementary material for: A Population-Based Approach to Study the Impact of PROP Perception on Food Liking in Populations along the Silk Road
Source: PLoS One. 2014 Mar 13;9(3):e91716. doi: 10.1371/journal.pone.0091716 (PMC3953580; doi:10.1371/journal.pone.0091716)
Supplement: Table S2 — The percentage of AVI/AVI, PAV/AVI and PAV/PAV subjects in the groups of NT (non taster), MT (medium taster) and ST (super taster) for each population. (DOC) [file pone.0091716.s003.doc]

| **TAS2R38 Genotype** | **PROP Status** | | |
| --- | --- | --- | --- |
|  | **NT** | **MT** | **ST** |
|  |  |  |  |
| *Georgia* |  |  |  |
| PAV/PAV | 25.0% | 60.7% | 14.3% |
| PAV/AVI | 34.7% | 51.0% | 14.3% |
| AVI/AVI | 89.7% | 7.7% | 2.6% |
|  |  |  |  |
| *Azerbaijan* |  |  |  |
| PAV/PAV | 23.1% | 46.1% | 30.8% |
| PAV/AVI | 38.5% | 57.7% | 3.84% |
| AVI/AVI | 71.4% | 14.3% | 14.3% |
|  |  |  |  |
| *Uzbekistan* |  |  |  |
| PAV/PAV | 33.3% | 41.7% | 25.0% |
| PAV/AVI | 28.3% | 54.3% | 17.4% |
| AVI/AVI | 80.0% | 5.0% | 15.0% |
|  |  |  |  |
| Kazakhstan |  |  |  |
| PAV/PAV | 6.7% | 66.7% | 26.6% |
| PAV/AVI | 17.9% | 60.7% | 21.4% |
| AVI/AVI | 85.7% | 14.3% | 0% |
|  |  |  |  |
| Tajikistan |  |  |  |
| PAV/PAV | 13.0% | 34.8% | 52.2% |
| PAV/AVI | 21.1% | 44.7% | 34.2% |
| AVI/AVI | 94.7% | 5.3% | 0% |
|  |  |  |  |
| Armenia |  |  |  |
| PAV/PAV | 3.2% | 45.2% | 51.6% |
| PAV/AVI | 12.0% | 42.0% | 46.0% |
| AVI/AVI | 66.7% | 25.0% | 8.3% |

**Table S2. The percentage of AVI/AVI, PAV/AVI and PAV/PAV subjects in the groups of NT (non tasters), MT (medium tasters) and ST (super tasters) for each population**
