# Supplementary material for: A Population-Based Approach to Study the Impact of PROP Perception on Food Liking in Populations along the Silk Road
Source: PLoS One. 2014 Mar 13;9(3):e91716. doi: 10.1371/journal.pone.0091716 (PMC3953580; doi:10.1371/journal.pone.0091716)
Supplement: Table S3 — Mean and standard deviation of each food included in the food liking questionnaire in the overall sample and in each population. (DOCX) [file pone.0091716.s004.docx]

|  | **All** | **Population** | | | | | |
| --- | --- | --- | --- | --- | --- | --- | --- |
| **Food** |  | *Georgia* | *Azerbaijan* | *Uzbekistan* | *Kazakhstan* | *Tajikistan* | *Armenia* |
| Liver | 3.3±1.5 | 3.1±1.3 | 2.8±1.7 | 3.8±1.2 | 3.1±1.4 | 4.0±1.4 | 2.7±1.7 |
| Dark chocolate | 3.7±1.4 | 3.7±1.4 | 3.0±1.5 | 3.9±1.3 | 3.6±1.4 | 3.9±1.4 | 3.9±1.5 |
| Milk chocolate | 4.3±1.1 | 4.3±1.0 | 3.9±1.2 | 4.5±1.0 | 4.2±1.1 | 4.2±1.4 | 4.6±1.0 |
| Coffee | 3.7±1.4 | 3.7±1.3 | 3.5±1.4 | 3.6±1.4 | 3.7±1.2 | 3.8±1.4 | 3.8±1.7 |
| Orange juice | 4.4±0.98 | 4.2±1.1 | 4.5±0.7 | 4.4±0.9 | 4.5±0.9 | 4.6±0.9 | 4.5±1.0 |
| Lemons | 3.8±1.3 | 3.6±1.2 | 4.0±1.3 | 3.8±1.2 | 3.6±1.1 | 4.0±1.4 | 3.9±1.5 |
| Sweet Bread | 3.9±1.3 | 3.6±1.3 | 3.8±1.4 | 4.0±1.2 | 3.9±1.1 | 4.3±1.2 | 3.9±1.4 |
| Red wine | 3.2±1.5 | 3.4±1.4 | 2.4±1.4 | 2.9±1.5 | 3.4±1.2 | 2.4±1.6 | 3.6±1.6 |
| White wine | 2.9±1.5 | 3.3±1.4 | 2.2±1.4 | 2.6±1.4 | 3.2±1.2 | 2.0±1.5 | 3.1±1.7 |
| Beer | 3.1±1.6 | 3.3±1.5 | 3.6±1.5 | 3.2±1.6 | 2.6±1.4 | 2.7±1.8 | 2.9±1.8 |
| Vodka | 2.3±1.4 | 2.4±1.4 | 2.7±1.6 | 2.0±1.4 | 2.2±1.1 | 1.8±1.5 | 2.3±1.5 |
| Pork | 3.6±1.5 | 4.0±1.0 | 2.0±1.3 | 2.6±1.6 | 2.6±1.3 | 2.1±1.7 | 4.4±1.2 |
| Ham | 3.8±1.4 | 3.9±1.1 | 2.7±1.5 | 3.5±1.5 | 3.2±1.4 | 3.2±1.7 | 4.3±1.4 |
| Mushrooms | 3.7±1.4 | 3.8±1.3 | 3.5±1.4 | 3.7±1.3 | 3.8±1.2 | 3.7±1.5 | 3.7±1.6 |
| Asparagus | 2.9±1.4 | 3.0±1.4 | 2.7±1.6 | 2.8±1.2 | 3.0±1.2 | 2.9±1.5 | 2.7±1.6 |
| Spinach | 3.4±1.5 | 3.3±1.3 | 3.3±1.4 | 3.9±1.4 | 2.9±1.3 | 3.6±1.5 | 3.4±1.6 |
| Cabbage | 3.9±1.2 | 3.4±1.1 | 3.7±1.2 | 3.8±1.1 | 3.9±1.1 | 4.3±1.2 | 4.2±1.3 |
| Garlic | 3.5±1.4 | 3.4±1.2 | 3.6±1.5 | 3.7±1.3 | 3.0±1.3 | 4.0±1.4 | 3.5±1.6 |
| Onion | 3.7±1.3 | 3.6±1.1 | 3.8±1.3 | 3.6±1.4 | 3.1±1.3 | 4.2±1.3 | 3.6±1.6 |
| Kilka | 3.4±1.4 | 3.1±1.3 | 3.3±1.4 | 3.8±1.4 | 3.3±1.2 | 3.8±1.5 | 3.3±1.6 |
| Olives | 3.5±1.5 | 3.1±1.5 | 3.2±1.5 | 3.2±1.4 | 3.6±1.3 | 3.8±1.5 | 4.2±1.4 |
| Artichokes | 2.6±1.4 | 2.3±1.2 | 2.1±1.3 | 2.7±1.3 | 2.7±1.1 | 3.3±1.6 | 2.3±1.4 |
| Ice cream | 4.5±0.95 | 4.4±0.9 | 4.3±0.9 | 4.5±0.9 | 4.3±1.0 | 4.6±1.0 | 4.5±1.0 |
| Cooked salami | 4.0±1.3 | 4.0±1.1 | 3.6±1.5 | 4.3±1.1 | 3.5±1.4 | 4.0±1.7 | 4.3±1.3 |
| Tea | 4.4±1.0 | 3.9±1.1 | 4.7±0.6 | 4.7±0.7 | 4.5±0.8 | 4.7±0.9 | 4.2±1.3 |
| Sardines | 3.8±1.3 | 3.6±1.2 | 3.7±1.3 | 4.2±1.0 | 3.7±1.1 | 4.1±1.4 | 3.4±1.6 |
| Chilli pepper | 2.9±1.6 | 3.2±1.3 | 2.6±1.5 | 2.5±1.5 | 2.7±1.3 | 3.2±1.8 | 2.8±1.7 |
| Cake | 4.2±1.1 | 4.1±1.2 | 4.1±1.2 | 4.5±0.9 | 4.2±1.2 | 4.5±1.0 | 4.3±1.3 |
| Lamb | 4.0±1.3 | 3.8±1.3 | 4.0±1.3 | 4.3±1.1 | 4.0±1.2 | 4.6±0.9 | 3.7±1.6 |
| Brandy | 2.7±1.5 | 3.4±1.4 | 2.1±1.5 | 2.2±1.4 | 2.5±1.3 | 2.6±1.7 | 2.4±1.6 |
| Peas | 3.5±1.4 | 2.9±1.2 | 3.9±1.1 | 3.6±1.3 | 3.3±1.2 | 4.0±1.4 | 3.7±1.5 |
| Beans | 3.6±1.3 | 3.6±1.1 | 3.8±1.3 | 3.7±1.4 | 3.1±1.2 | 3.8±1.5 | 3.5±1.5 |
| Broad beans | 3.6±1.4 | 3.3±1.3 | 3.7±1.4 | 3.5±1.4 | 3.2±1.1 | 3.6±1.5 | 4.0±1.4 |
| Tomato | 4.6±0.80 | 4.4±0.9 | 4.1±1.1 | 4.7±0.6 | 4.6±0.6 | 4.8±0.6 | 4.7±0.7 |
| Turnip | 3.2±1.4 | 2.8±1.3 | 3.7±1.1 | 3.2±1.4 | 3.1±1.1 | 3.6±1.5 | 3.2±1.5 |
| Walnuts | 4.5±0.88 | 4.4±0.9 | 4.6±0.7 | 4.5±0.9 | 4.5±0.7 | 4.7±0.9 | 4.7±0.9 |
| Pomegranate | 4.6±0.83 | 4.4±0.8 | 4.5±0.7 | 4.7±0.7 | 4.5±0.9 | 4.7±0.8 | 4.7±0.9 |
| Cucumber | 4.4±0.89 | 4.2±0.9 | 4.3±0.9 | 4.6±0.7 | 4.4±0.8 | 4.6±1.0 | 4.6±0.9 |
| Milk | 3.9±1.4 | 3.5±1.4 | 3.8±1.4 | 4.0±1.3 | 4.4±0.9 | 4.4±1.2 | 3.8±1.5 |
| Yogurt | 3.9±1.4 | 3.6±1.4 | 3.7±1.4 | 4.2±1.2 | 4.3±0.9 | 4.5±1.0 | 3.5±1.7 |
| Sheep cheese | 3.6±1.4 | 3.3±1.4 | 3.9±1.4 | 3.7±1.3 | 3.9±1.1 | 4.2±1.2 | 3.3±1.7 |
| Melted cheese | 3.8±1.4 | 3.2±1.4 | 3.2±1.5 | 4.0±1.2 | 4.1±1.1 | 4.4±1.0 | 3.7±1.6 |
| Cottage cheese | 3.7±1.4 | 3.4±1.3 | 3.2±1.5 | 3.8±1.4 | 4.5±0.7 | 4.1±1.3 | 3.5±1.7 |
| Kurut | 3.6±1.4 | 2.8±1.2 | 2.6±1.3 | 4.0±1.3 | 3.9±1.0 | 4.3±1.1 | 2.8±1.6 |
| Smoked cheese | 3.9±1.3 | 4.1±1.0 | 3.0±1.2 | 3.8±1.3 | 3.9±1.1 | 4.1±1.3 | 4.0±1.4 |
| Sulguni | 4.0±1.3 | 4.2±1.0 | 3.8±1.4 | 3.3±1.4 | 3.1±1.1 | 3.0±1.6 | 4.5±1.0 |
| Sweet ricotta | 3.4±1.5 | 3.2±1.4 | 3.1±1.3 | 3.8±1.3 | 4.2±0.9 | 3.9±1.5 | 2.8±1.6 |
| Salt ricotta | 3.2±1.5 | 3.1±1.3 | 3.6±1.3 | 3.2±1.4 | 3.6±1.2 | 3.2±1.6 | 2.8±1.7 |
| Buckwheat | 3.6±1.4 | 3.3±1.2 | 3.6±1.5 | 3.9±1.3 | 3.6±1.2 | 3.8±1.4 | 3.5±1.6 |
| Dried biscuits | 4.1±1.1 | 4.0±1.1 | 4.0±1.0 | 4.2±1.1 | 3.7±1.1 | 4.3±1.1 | 4.2±1.3 |
| Cream biscuits | 4.0±1.2 | 4.0±1.0 | 4.0±1.2 | 4.0±1.1 | 3.8±1.1 | 4.3±1.2 | 4.1±1.4 |
| Eggplant | 3.8±1.4 | 3.8±1.3 | 3.3±1.5 | 3.7±1.4 | 3.4±1.1 | 3.7±1.6 | 4.2±1.2 |
| Cooked carrots | 3.2±1.5 | 3.0±1.3 | 3.7±1.5 | 3.2±1.5 | 3.3±1.2 | 3.8±1.7 | 2.7±1.5 |
| Raw carrots | 4.2±1.1 | 3.7±1.3 | 4.2±1.0 | 4.0±1.2 | 4.1±0.9 | 4.6±0.8 | 4.6±1.0 |
| Vinegar | 2.5±1.5 | 2.8±1.4 | 2.1±1.2 | 2.5±1.5 | 2.2±1.2 | 2.5±1.6 | 2.6±1.6 |
| Mutton | 3.9±1.4 | 3.4±1.4 | 4.1±1.3 | 4.1±1.3 | 3.9±1.3 | 4.7±0.7 | 3.4±1.7 |
| Almonds | 4.3±1.1 | 3.7±1.2 | 4.0±1.3 | 4.6±0.8 | 4.2±1.0 | 4.5±1.0 | 4.6±0.9 |
| Cherry | 4.6±0.83 | 4.3±0.9 | 4.7±0.5 | 4.6±0.8 | 4.4±0.9 | 4.6±0.8 | 4.8±0.8 |
| Plum | 4.3±0.99 | 4.1±1.0 | 4.3±0.9 | 4.1±1.1 | 4.2±1.0 | 4.5±0.9 | 4.6±0.9 |
| Apple | 4.7±0.73 | 4.3±1.0 | 4.7±0.6 | 4.8±0.6 | 4.6±0.7 | 4.9±0.5 | 4.8±0.7 |
| Honey | 4.2±1.1 | 4.0±1.1 | 4.4±1.1 | 4.4±1.0 | 4.3±1.1 | 4.3±1.3 | 4.2±1.2 |
| Jam | 4.1±1.1 | 3.8±1.1 | 4.1±1.2 | 4.3±1.0 | 4.0±0.9 | 4.3±1.2 | 4.1±1.3 |
| Blackberry | 4.2±1.1 | 4.0±1.1 | 3.9±1.2 | 4.3±1.1 | 4.1±1.0 | 4.3±1.2 | 4.5±1.1 |
| Dried Fruits | 4.2±1.2 | 3.8±1.1 | 3.8±1.4 | 4.3±1.1 | 4.1±1.2 | 4.5±1.1 | 4.4±1.1 |
| Strawberry | 4.7±0.70 | 4.5±0.8 | 4.8±0.4 | 4.7±0.7 | 4.8±0.5 | 4.7±0.8 | 4.9±0.5 |
| Prunes | 4.2±1.1 | 4.1±1.1 | 4.4±0.9 | 4.1±1.2 | 4.3±1.0 | 4.3±1.0 | 4.4±1.2 |
| Pepper | 3.8±1.3 | 3.3±1.3 | 3.6±1.3 | 3.6±1.3 | 3.8±1.1 | 4.2±1.2 | 4.0±1.4 |
| Adgika | 3.6±1.4 | 3.7±1.3 | 3.5±1.4 | 3.4±1.4 | 3.1±1.3 | 3.8±1.4 | 3.7±1.6 |
| Dill | 3.7±1.4 | 3.2±1.3 | 3.8±1.1 | 3.7±1.3 | 3.6±1.3 | 4.4±1.1 | 3.6±1.6 |
| Barley | 2.8±1.4 | 2.7±1.3 | 2.3±1.5 | 2.9±1.3 | 2.8±1.1 | 3.7±1.4 | 2.5±1.4 |
| Butter | 4.0±1.2 | 3.9±0.9 | 4.2±1.2 | 4.2±1.1 | 3.7±1.2 | 4.6±0.8 | 3.6±1.6 |
| Watermelon | 4.8±0.64 | 4.6±0.7 | 4.5±0.9 | 4.8±0.6 | 4.8±0.4 | 4.9±0.3 | 4.8±0.7 |
| Cranberry | 3.6±1.4 | 3.5±1.3 | 3.7±1.3 | 3.6±1.3 | 3.7±1.3 | 4.2±1.3 | 3.9±1.4 |
| Black raspberry | 3.6±1.4 | 3.4±1.4 | 3.6±1.2 | 3.6±1.5 | 3.7±1.2 | 3.9±1.3 | 3.6±1.5 |
| Banana | 4.3±1.2 | 4.2±1.5 | 4.3±1.1 | 4.2±1.3 | 4.5±0.8 | 4.0±1.4 | 4.5±1.1 |
| Rice | 4.2±1.1 | 3.7±1.1 | 4.1±1.1 | 4.6±0.8 | 4.2±0.9 | 4.7±0.8 | 4.1±1.4 |
| Beet | 3.5±1.4 | 3.1±1.3 | 3.1±1.4 | 3.9±1.3 | 3.4±1.1 | 3.9±1.3 | 3.5±1.6 |
| Whipped cream | 3.9±1.4 | 3.7±1.2 | 4.0±1.2 | 4.0±1.2 | 3.8±1.3 | 4.2±1.3 | 3.6±1.7 |
| Melon | 4.4±1.1 | 4.3±1.0 | 4.0±1.3 | 4.8±0.5 | 4.6±0.7 | 4.8±0.7 | 4.0±1.5 |

**Table S3. Mean and standard deviation of each food included in the food liking questionnaire in the overall sample and in each population.**
