# Supplementary material for: A Population-Based Approach to Study the Impact of PROP Perception on Food Liking in Populations along the Silk Road
Source: PLoS One. 2014 Mar 13;9(3):e91716. doi: 10.1371/journal.pone.0091716 (PMC3953580; doi:10.1371/journal.pone.0091716)
Supplement: Table S4 — Mean and standard deviation of food liking groups in each population. (DOCX) [file pone.0091716.s005.docx]

|  | **All** | **PROP status** | | |
| --- | --- | --- | --- | --- |
|  |  | **NT** | **MT** | **ST** |
|  |  | (mean±sd) | | |
| **Georgia** |  |  |  |  |
| sweet | 3.9±0.7 | 4.1±0.6 | 4.0±0.5 | 3.3±1.2 |
| fruits | 4.1±0.8 | 4.2±0.7 | 4.3±0.6 | 3.3±1.3 |
| vegetables | 3.1±0.8 | 3.1±0.8 | 3.1±0.8 | 3.0±0.8 |
| alcohol | 3.1±1.1 | 3.1±1.1 | 3.1±1.1 | 3.1±1.1 |
| condiments | 3.4±0.8 | 3.4±0.7 | 3.5±0.8 | 2.9±0.8 |
| **Azerbaijan** |  |  |  |  |
| sweet | 4.0±0.7 | 3.9±0.8 | 4.1±0.7 | 4.2±0.6 |
| fruits | 4.5±0.5 | 4.5±0.5 | 4.4±0.5 | 4.5±0.5 |
| vegetables | 3.5±1.0 | 3.5±0.6 | 3.5±0.6 | 3.7±1.2 |
| alcohol | 2.5±1.2 | 2.5±1.4 | 2.6±1.1 | 2.3±0.8 |
| condiments | 3.4±0.8 | 3.5±0.8 | 3.3±0.7 | 3.7±0.9 |
| **Uzbekistan** |  |  |  |  |
| sweet | 4.3±0.7 | 4.2±0.7 | 4.2±0.7 | 4.5±0.6 |
| fruits | 4.3±0.7 | 4.3±0.7 | 4.2±0.7 | 4.5±0.6 |
| vegetables | 3.4±0.9 | 3.5±0.9 | 3.1±0.9 | 3.9±0.9 |
| alcohol | 2.7±1.2 | 2.8±1.1 | 2.7±1.2 | 2.4±1.3 |
| condiments | 3.5±0.9 | 3.4±0.7 | 3.5±0.9 | 3.6±1.0 |
| **Kazakhstan** |  |  |  |  |
| sweet | 4.1±0.6 | 4.0±0.7 | 4.0±0.6 | 4.3±0.6 |
| fruits | 4.3±0.6 | 4.2±0.6 | 4.4±0.5 | 4.3±0.5 |
| vegetables | 3.2±0.8 | 3.0±0.9 | 3.4±0.7 | 2.9±0.9 |
| alcohol | 2.8±0.9 | 2.4±1.0 | 2.9±0.8 | 3.2±0.8 |
| condiments | 3.2±0.9 | 2.9±0.9 | 3.5±0.7 | 2.9±1.1 |
| **Tajikistan** |  |  |  |  |
| sweet | 4.3±0.7 | 4.5±0.5 | 4.3±0.7 | 4.1±0.9 |
| fruits | 4.4±0.8 | 4.4±0.8 | 4.3±0.8 | 4.5±0.8 |
| vegetables | 3.7±1.0 | 4.0±1.0 | 3.8±1.0 | 3.3±1.1 |
| alcohol | 2.4±1.4 | 1.8±1.2 | 2.7±1.7 | 2.7±1.3 |
| condiments | 3.8±0.9 | 3.9±0.8 | 3.9±1.0 | 3.6±1.0 |
| **Armenia** |  |  |  |  |
| sweet | 4.1±0.8 | 4.3±0.7 | 4.0±0.9 | 4.2±0.8 |
| fruits | 4.4±0.7 | 4.5±0.8 | 4.3±0.7 | 4.5±0.7 |
| vegetables | 3.4±1.0 | 3.5±1.0 | 3.3±1.1 | 3.4±0.9 |
| alcohol | 2.9±1.2 | 3.0±1.3 | 3.0±1.1 | 2.8±1.1 |
| condiments | 3.5±0.9 | 3.8±0.7 | 3.4±0.9 | 3.4±0.8 |

**Table S4. Mean and standard deviation of food liking groups in each population.** In the table are reported mean and standard deviation in the overall sample (All column) and according to taster status (PROP status column). NT = non taster, MT = medium taster, ST =super taster.
