# Supplementary material for: A Population-Based Approach to Study the Impact of PROP Perception on Food Liking in Populations along the Silk Road
Source: PLoS One. 2014 Mar 13;9(3):e91716. doi: 10.1371/journal.pone.0091716 (PMC3953580; doi:10.1371/journal.pone.0091716)
Supplement: Table S5 — Pair-wise distance matrix based on food liking. (DOCX) [file pone.0091716.s006.docx]

|  | **Uzbekistan** | **Armenia** | **Georgia** | **Azerbaijan** | **Kazakhstan** | **Tajikistan** |
| --- | --- | --- | --- | --- | --- | --- |
| **Uzbekistan** | 0 | 10 | 23 | 3 | 1 | 4 |
| **Armenia** |  | 0 | 27 | 11 | 17 | 13 |
| **Georgia** |  |  | 0 | 5 | 8 | 39 |
| **Azerbaijan** |  |  |  | 0 | 3 | 11 |
| **Kazakhstan** |  |  |  |  | 0 | 15 |
| **Tajikistan** |  |  |  |  |  | 0 |

**Table S5. Pair-wise distance matrix based on food liking**
