# Supplementary material for: A Population-Based Approach to Study the Impact of PROP Perception on Food Liking in Populations along the Silk Road
Source: PLoS One. 2014 Mar 13;9(3):e91716. doi: 10.1371/journal.pone.0091716 (PMC3953580; doi:10.1371/journal.pone.0091716)
Supplement: Table S6 — Pair-wise distance matrix based on PROP status. (DOCX) [file pone.0091716.s007.docx]

|  | **Uzbekistan** | **Armenia** | **Georgia** | **Azerbaijan** | **Kazakhstan** | **Tajikistan** |
| --- | --- | --- | --- | --- | --- | --- |
| **Uzbekistan** | 0 | 12.4 | 3.7 | 0.57 | 1.6 | 3.7 |
| **Armenia** |  | 0 | 31.4 | 9.7 | 7.9 | 4.6 |
| **Georgia** |  |  | 0 | 2.2 | 6.0 | 13.7 |
| **Azerbaijan** |  |  |  | 0 | 0.53 | 4.8 |
| **Kazakhstan** |  |  |  |  | 0 | 5.4 |
| **Tajikistan** |  |  |  |  |  | 0 |

**Table S6. Pair-wise distance matrix based on PROP status**
